# Bacterial Indole-3-Acetic Acid Influences Soil Nitrogen Acquisition in Barley and Chickpea

**DOI:** 10.3390/plants10040780

**Published:** 2021-04-16

**Authors:** Shraddha Gang, Sheetal Sharma, Meenu Saraf, Martin Buck, Jorg Schumacher

**Affiliations:** 1 Department of Life Science, Faculty of Natural Sciences, Imperial College, London SW7 2AZ, UK; s.gang@imperial.ac.uk (S.G.); m.buck@imperial.ac.uk (M.B.); 2 Department of Microbiology and Biotechnology, School of Sciences, Gujarat University, Ahmedabad 380009, India; sheetumsharma2961994@gmail.com

**Keywords:** nitrogen use efficiency, nitrogen uptake efficiency, nitrogen utilization efficiency, *Klebsiella*, indole acetic acid, root architecture

## Abstract

Farming of barley and chickpea is nitrogen (N) fertilizer dependent. Using strategies that increase the nitrogen use efficiency (NUE) and its components, nitrogen uptake efficiency (NUpE) and nitrogen utilization efficiency (NUtE) would reduce the N fertilizer application in the soil and its adverse environmental effects. We evaluated the effects of three different strains of diazotroph *Klebsiella* (*K.p.* SSN1, *K.q.* SGM81, and *K.o.* M5a1) to understand the role of biological nitrogen fixation (BNF) and bacterial indole-3-acetic acid (IAA) on NUE of the plants. A field study revealed that *K.p.* SSN1 results in profound increment of root surface area by eightfold and threefold compared to uninoculated (control) in barley and chickpea, respectively. We measured significant increase in the plant tissue nitrogen, chlorophyll content, protein content, nitrate reductase activity, and nitrate concentration in the inoculated plants (*p* ≤ 0.05). Treated barley and chickpea exhibited higher NUE and the components compared to the control plants (*K.p.* SSN1 ≥ *K.q.* SGM81> *K.o.* M5a1). Specifically, *K.q.* SGM81 treatment in barley increased NUpE by 72%, while in chickpea, *K.p.* SSN1 increased it by 187%. The substantial improvement in the NUpE and NUE by the auxin producers *K.p.* SSN1 and *K.q.* SGM81 compared with non-auxin producer *K.o.* M5a1 was accompanied by an augmented root architecture suggesting larger contribution of IAA over marginal contribution of BNF in nitrogen acquisition from the soil.

## 1. Introduction

With rapid and continuing growth of the world’s population and the resulting demand in food production, excessive use of chemical fertilizer is degrading the agricultural soil health. Amongst many others, lack of nitrogen is classified as an important abiotic stress factor [1] and its deficiency in soil negatively affects many physiological processes in a plant. To overcome this shortage, different forms of nitrogen are applied to the soil as nitrogen fertilizers. According to an FAO report, the worldwide consumption of nitrogen fertilizer in 2020 was 108.74 million tons, which is expected to reach 111.9 million tons in 2022 [2]. One alternative solution to replace or minimize the use of nitrogen fertilizers is to better harness the biological nitrogen fixation (BNF) in which nitrogenase enzymes from microorganisms can provide fixed nitrogen to plants [3]. However, the practice of using a microbial strategy to fix nitrogen is limited due to a lower output in terms of yield, hence the use of chemical nitrogen sources persists in agriculture [4]. One problem lies in the fact that only around 30% of the applied nitrogen is utilized by the crop plants and the remaining unutilized nitrogen causes severe environmental pollution [5]. Increased nitrogen use efficiency (NUE) could alleviate these environmental impacts and is generally divided into two phases; a. the nitrogen uptake efficiency (NUpE) of the plant that involves assimilation, and b. nitrogen utilization efficiency (NUtE) that involves remobilization of nitrogen [6].

The use of plant growth promoting microorganisms (PGPMs) could be a feasible solution to improve NUE. The few main mechanisms of the rhizosphere that influence N-uptake efficiency are: root size morphology, root N transporter system, and interaction of root–microorganisms such as plant growth-promoting bacteria (PGPB) [7]. Many PGPBs are reported to positively improve root system development by manipulating plant hormones such as auxins, cytokinins, or gibberellins [8,9], and hence the acquisition of some nutrients. NO_3_^−^ and NH_4_^+^ uptake systems may be enhanced by the interaction with arbuscular mycorrhizal fungi (AMF), PGPB, and some organic acids [7,10]. The pursuit of BNF, especially in cereals, started long ago with isolation of nitrogen-fixing bacteria from different cereal plants [11]. Few studies have also focused on the appearance of diazotrophs in association with cereal crops and evidence of nitrogen supply [12]. One such example of a bacterial diazotroph is *Klebsiella.* It has been dominant and widely pervasive in the rhizosphere of a variety of plants such as maize [13], wheat [14,15], and sugarcane [16]. Here we explore the potential of highly ubiquitous *Klebsiella* as a tool to improve plant NUE. We hypothesized two bacterial mechanisms that might play an important role. First, the nitrogen-fixing ability of the wild type strains, and second, the auxin (IAA) production. The latter will influence the root structure and thus help in acquisition of soil nitrogen (no chemical nitrogen fertilizer supplements in the present case). In proteobacteria, nitrogen fixation genes (*nif*) are generally transcribed by the alternative s^54^ RNA-polymerase under nitrogen limiting conditions and regulated by the enhancer binding protein NifA [17]. Auxin production depends on the expression of *ipdC*, coding for thiamindiphosphate-dependent indolepyruvate decarboxylase, which was reported to also be s^54^-dependent in the diazotrophic *Azospirillum brasilense* [18] and the non-diazotrophic *Enterobacter cloacae* [19].

To understand which out of the two aforementioned mechanisms has larger impact, the effects of three different strains of *Klebsiella* (from different origin) on a model monocot *Hordeum vulgare * (barley) and a dicot legume *Cicer arietinum* (chickpea) were analyzed. The selected wildtype strains were *Klebsiella pneumoniae* SSN1, *Klebsiella quasipneumoniae* SGM81, and *Klebsiella oxytoca* M5a1, which hereafter will be represented as *K.p*. SSN1, *K.q.* SGM81, and *K.o.* M5a1, respectively. The choice of strains was made considering their ability of BNF (*K.p*. SSN1, *K.q.* SGM81, and *K.o.* M5a1), IAA production (*K.p*. SSN1, *K.q.* SGM81), and a negative control (Δ*nifH)* which is a nitrogenase minus variant of wild type *K.o.* M5a1 lacking both BNF and IAA production. Thus, we determined if: (1) wildtype *Klebsiella* strains (test strains) positively affect the barley and chickpea growth parameters, and (2) the enhanced structural context of root triggers the plant’s NUE and its components.

## 2. Results

### 2.1. Characterization for Biological Nitrogen Fixation and Phytohormone Production in Klebsiella Strains

The strains *K.q.* SGM81, *K.o.* M5a1, and *K.p.* SSN1 were based on the prediction of BNF and IAA production activities from their whole genome sequence data (details described in Section 4). The presence of *nif* gene cluster and *ipdC* gene pathway in all three genomes indicate the BNF and IAA production activities in *K.p.* SSN1, *K.q.* SGM81, and *K.o.* M5a1 strains.

**Figure 1:**
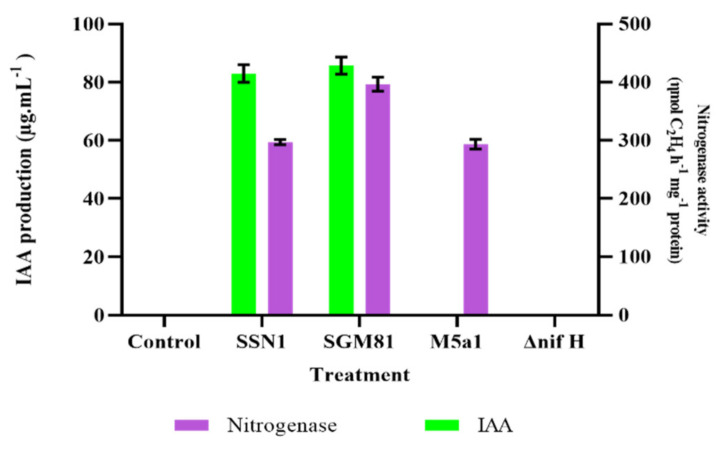
Indole-3-acetic acid (IAA) production and acetylene reduction in four strains. Control is the value of uninoculated media. Bars represents mean values of replicates. Error bars represent standard error from the mean (*n* = 5).

First, we screened all the *Klebsiella* strains (*K.p.* SSN1, *K.q.* SGM81, *K.o.* M5a1, M5a1Δ*nifH*) for their nitrogenase activity and IAA production ability in batch culture. The results of acetylene reduction assay (ARA) showed that the wild type strains exhibit considerable nitrogenase activity, indicating the active expression of *nifH* gene. As shown in Figure 1, *K.q.* SGM81 showed maximum, i.e., 396.5 nmol, C_2_H_4_ h^−1^ mg protein^−1^ of nitrogenase activity, followed by *K.p.* SSN1 (297.0 nmol C_2_H_4_ h^−1^ mg protein^−1^). Nitrogenase activity of *K.o.* M5a1 was recorded as 293.5 nmol C_2_H_4_ h^−1^ mg protein^−1^. As expected, no nitrogenase activity was detected in M5a1Δ*nifH*.

Next, we quantified the tryptophan-induced indole-3-acetic acid using the Salkowski method. The results of this assay are presented in Figure 1. The highest IAA production was seen in *K.q.* SGM81 (85.75 µg mL^−1^) followed by *K.p.* SSN1. *K.o.* M5a1 and its *nifH* mutant did not produce any IAA, suggesting a non-functional *ipdC* gene. A Clustal O multiple sequence alignment of *ipdC* sequences of the three strains (Figure S1) was carried out, showing pairwise percent identities 85.17%, 85.35%, and 99.64% similarity in protein sequence between *K.o.* M5a1–*K.q.* SGM81, between *K.o.* M5a1–*K.p.* SSN1, and between *K.q.* SGM81–*K.p.* SSN1, respectively. However, the amino acid sequence alignment of *ipdC* of M5a1, SGM81, and SSN1 (Figure S1) shows 100% identity of known highly conserved residues involved in Mg^2+^ binding (red) and thiamine pyrophosphate binding (blue highlighted) with the functional *ipdC* of *Enterobacter cloacae*, for which the structure and function has been detailed. Therefore, there is no obvious structure/function rational from the *K.o.* M5a1 *ipdC* coding sequence to explain the lack of *K.o.* M5a1 to produce IAA. Next, we inspected the promoter regions of *K.q.* SGM81 and *K.o.* M5a1. Recall that *ipdC* transcription was proposed to be s^54^-dependent in *Enterobacter cloacae* and is s^54^-dependent diazotrophic *Azospirillum brasilense* [18]. We show that *K.q.* SGM81 has two near perfect s^54^ promoter sites at and around consensus the −24 and −12 sites from the transcriptional start with the required distance between them, whereas *K.o.* M5a1 shows no good s^54^ promoter sites (Figure S2). These finding suggest that the *K.o.* M5a1 ipdC s^54^ promoter is likely to have been corrupted. This seems feasible, given that *K.o.* M5a1 has been a decade-long laboratory strain.

### 2.2. IAA Biosynthesis in Klebsiella Contributes to Enhanced Root Traits

To understand the effect of diazotrophs on the root system of barley and chickpea, matured plants were harvested after 90 days (Figure S3) and roots were gently washed with water to remove adhered soil. Washed and drained-to-dry roots were then studied for six parameters, namely root length (RL), root fresh weight (RW), root dry weight (RDW), root diameter (Rootdia), root surface area (RSA), and the number of secondary roots (NSR). The results are described in Table 1. In general, the structural parameters of root in both plant types were positively altered by the wildtype strains. In barley, all the treated plants except Δ*nifH* showed superior phenotype than the control plants. Amongst all the traits, RSA and NSR were the most affected parameters. Compared to control, *K.p.* SSN1 showed profound effects on all the structural traits, specifically with eightfold and threefold increment in the RSA of barley and chickpea, respectively. The inoculation of chickpea with *K.q.* SGM81 resulted in maximum NSR increased by 113% and 60% than the control in barley and chickpea, respectively. The overall impact of different treatments in terms of their positive effect on barley and chickpea root can be summarized as *K.p.* SSN1 > *K.q.* SGM81 > *K.o.* M5a1 > control > Δ*nifH,* as shown in Table 1. Compared to the non-inoculated, plants treated with the *nifH* mutant showed a decrease in the root traits. Since M5a1 Δ*nifH* has neither beneficial IAA production capacity nor fixes nitrogen, the most likely explanation is that the high titer of M5a1 Δ*nifH* may partly exclude other (e.g., IAA microbes) soil-endogenous soil microbes in the rhizosphere from providing beneficial services to the plant. Plants under normal growth conditions establish a mutualistic relation with the soil flora, which could have been affected due to abundance of *nifH* mutant cells. Taken together, these results imply that *Klebsiella* strains capable of auxin production (*K.p.* SSN1 and *K.q.* SGM81) could largely be promoting enhanced root growth and hence more nutrient access compared to non-auxin producers (*K.o.* M5a1 and Δ*nifH*).

### 2.3. Klebsiella Improves Shoot Phenotype and Biochemical Profiling

**Figure 2:**
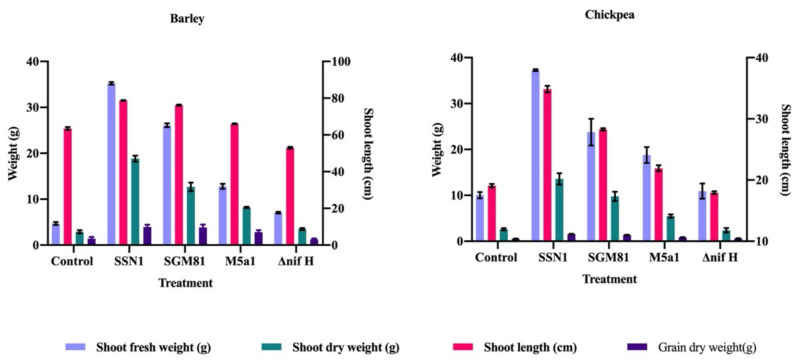
Effect of inoculation on barley and chickpea shoot traits from left to right. Shoot traits include shoot length, shoot fresh weight, shoot dry weight, and grain weight. Bars represent mean values of replicates. Error bars represent standard error from the mean (*n* = 6).

To validate the positive effect of diazotrophs on aerial parts of the host plants, we assessed the effect of bioinoculation on structural and biochemical traits of the shoot system. Four structural traits, shoot fresh weight (SW), shoot dry weight (SDW), shoot length (SL), and grain weight (GW), were measured. Here, GW was a crucial parameter reflecting the yield of a crop. It was measured as grain weight (g) per pod. The response was highly significant in *K.p.* SSN1 and *K.q.* SGM81 treatment in both plant varieties. Amongst all, *K.p.* SSN1 treatment showed the highest SL, SW, SDW, and GW, irrespective of monocot or dicot host plant (Figure 2). *K.p.* SSN1 resulted in 181.7% and 198% increase while *K.q.* SGM81 resulted in 174% and 155.2% increase in GW in barley and chickpea, respectively, over control plants. Compared to control plants, *K.o.* M5a1 resulted in 100% higher barley GW and 57.6% higher chickpea GW. Effect of Δ*nifH* inoculation was non-significant on SDW of chickpea and on GW of both barley and chickpea.

Next, we examined several biochemical characteristics of shoot, chlorophyll content (Cl), protein content (PR), nitrate reductase activity (NR), nitrate (NT) concentration, and total aboveground nitrogen (ABN). To study biochemical characteristics of plant post-uprooting, it was washed and was air-dried or kept fresh as required. Table 2 represents the results of all biochemical traits. In comparison to control plants, content of all parameters was improved in plants treated with wild type *Klebsiella* strains. For Cl, *K.p.* SSN1 showed 41.7% and 106.2% higher content than control in barley and chickpea, respectively, also being the highest among all other treatments. Estimation of total PR revealed that in barley, the highest PR content was in *K.p.* SSN1-treated plants, whereas in chickpea, *K.q.* SGM81 resulted in the highest protein with 51% increment compared to control. NR enzyme activity of *K.p.* SSN1 and *K.q.* SGM81-treated barley was almost similar. In chickpea, *K.o.* M5a1-treated plants resulted in 64.4% higher NR activity, which also was the highest among all other chickpea treatments. The highest NT was quantified in *K.p.* SSN1 plants of both varieties followed by *K.q.* SGM81 in barley and *K.o.* M5a1 in chickpea. Lastly, *K.q.* SGM81 resulted in the highest plant nitrogen in barley and *K.p.* SSN1 in chickpea, as shown in Table 2. The *ΔnifH* treatment failed to show any significant increase in the biochemical content above control plants. This demonstrates the failure of the *ΔnifH Klebsiella* in promoting plant growth. The improvement in all the biochemical traits in *K.p.* SSN1 and *K.q.* SGM81, but not *K.o.* M5a1, clearly demonstrates that a coupled effect of root growth-promoting traits (auxin production) and nitrogen-fixing ability of *K.p.* SSN1 and *K.q.* SGM81 is more vital than nitrogen-fixing ability alone of *K.o.* M5a1.

Collectively, from the above results, we infer that *Klebsiella* spp. contributed towards higher yield and improved biochemical characteristics, specifically those related to nitrogen accumulation or/and mobilization in barley and chickpea plants.

### 2.4. Klebsiella Demonstrates Effective Mobilization and Use of Nitrogen in Barley and Chickpea

To evaluate the nitrogen mobilization ability of differently treated plants, NupE and NUtE were calculated. NUpE is the very first mechanism taking place when plant root comes in contact with the soil nitrogen. Once taken up, nitrogen is then transported to aerial parts of the plant via xylem and redistributed in the shoot system referring to NUtE. The total nitrogen quantification in the above section reflects the higher potential of *K.p.* SSN1 and *K.q.* SGM81 to yield better grain quality and improved NUE as compared to *K.o.* M5a1.

**Figure 3:**
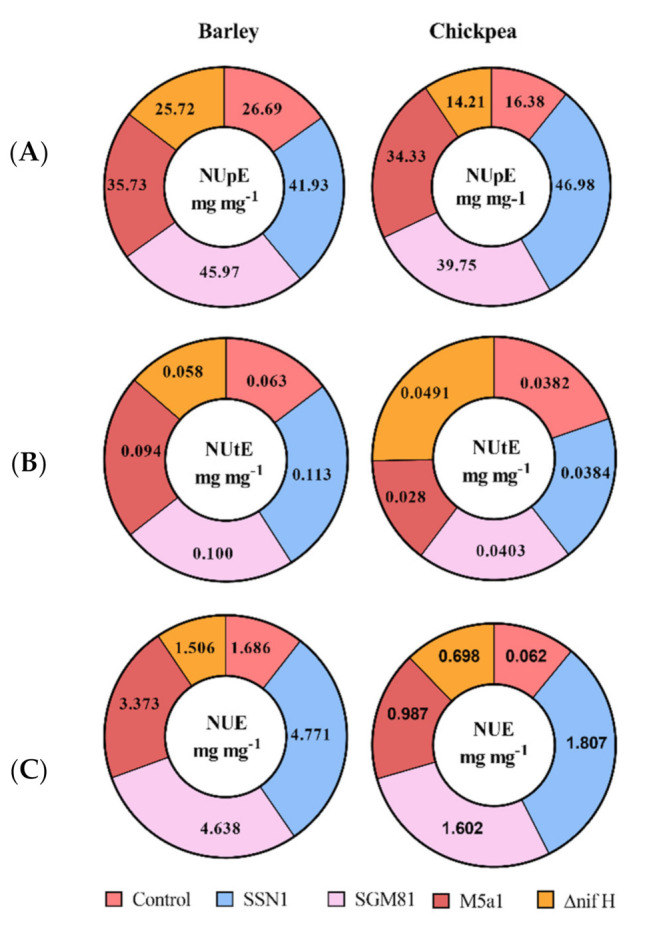
Nitrogen use efficiency and its components in barley and chickpea from left to right. (**A**) represents effect of inoculation on nitrogen uptake efficiency (NUpE), (**B**) represents effect of inoculation on nitrogen utilization efficiency (NUtE), (**C**) represents effect of inoculation on nitrogen use efficiency (NUE). Control is the value in uninoculated plants.

**Figure 4:**
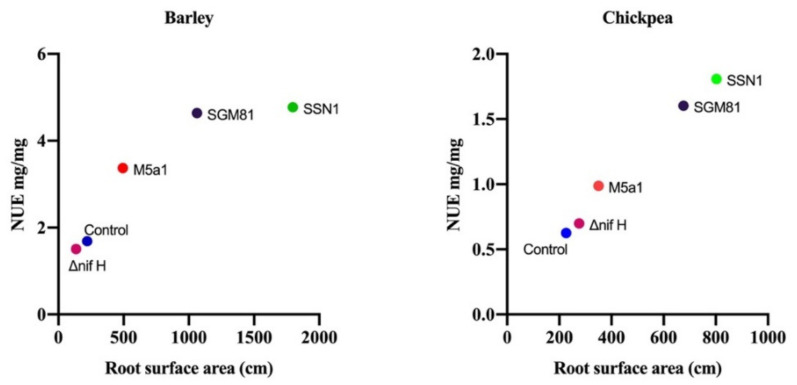
Correlation between root surface area (RSA) and nitrogen use efficiency (NUE) in barley and chickpea from left to right. The plot represents the increase in NUE with the increase in root surface area.

In general, as shown in Figure 3, NUE, NUpE, and NUtE in barley is significantly higher than that in chickpea. In barley, NUpE was the highest in *K.q.* SGM81 (72% higher than control) whereas NUpE of chickpea was maximum (187% higher than control) in *K.p.* SSN1 plants. The Δ*nifH* plants exhibited the lowest NUpE in both barley and chickpea. *K.p.* SSN1 treatment also showed maximum positive effect on NUtE and NUE of both barley and chickpea, followed by *K.q.* SGM81. The Δ*nifH* showed a little higher (28%) NUtE in chickpea plants than control. Interestingly, *K.o.* M5a1, which was positive for BNF, consistently followed *K.p.* SSN1 and *K.q.* SGM81 in terms of its positive effect compared to control. The correlation plot between NUE and RSA is shown in Figure 4. It is evident that increase in NUE is congruent to the increase in RSA of the roots.

Both barley and chickpea had the similar influence of RSA on NUE, showing positive correlation between the two. This result suggests that stronger root establishment in the soil gives the plant an improved access to soil nitrogen.

### 2.5. Principal Component Analysis

**Figure 5:**
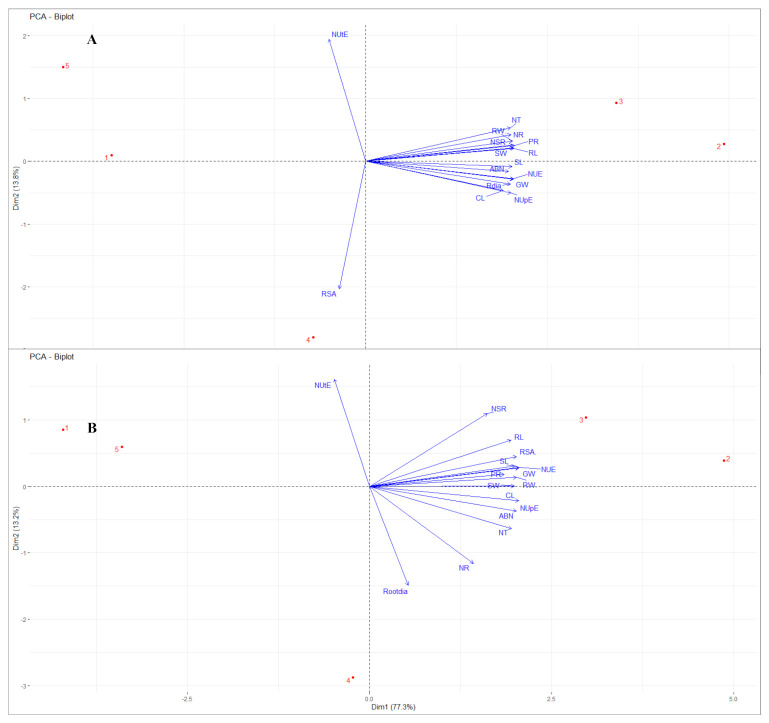
Principal component analysis (PCA) biplot for barley (**A**) and chickpea (**B**). Biplot represents correlation between plant traits with NUpE, NUtE, and NUE in different treatments. Red dots represent overall effect of all five treatments, viz., 1 = control, 2 = *K.p.* SSN1, 3 = *K.q.* SGM81, 4 = M5a1, and 5 = Δ*nif H*. Vectors in blue represent plant traits. NSR: number of secondary roots, RL: root length, RSA: root surface area, Rootdia: root diameter, SL: shoot length, SW: shoot fresh weight, RW: root fresh weight, GW: grain weight, PR: protein, Cl: chlorophyll, ABN: aboveground nitrogen, NR: nitrate reductase, NT: nitrate, NUtE: nitrogen utilization efficiency, NUpE: nitrogen uptake efficiency, NUE: nitrogen use efficiency.

A principal component analysis (PCA) biplot was carried out for all the traits studied. The association of traits among first and second principal component is shown in Figure 5. In barley (Figure 5A), principal component 1 (dimension 1) showed variation up to 82.2% comprising root structure parameters including RW, RL, and root diameter, all shoot morphological parameters, and biochemical traits, viz., NR, Cl, PR, and ABN in presence of strains *K.p.* SSN1 and *K.q.* SGM81. RSA and NUtE lies on PC2 (dimension 2) showing negative correlation with 13.8% variation with other traits. Principal component analysis of chickpea is shown in Figure 5B, where both principal components explained about 90% of the total variation. The principal component 1 (dimension 1) spanned all the traits but NUtE. RSA in chickpea, unlike barley, is loaded on PC1 showing positive correlation with NUE, NUpE, and all other traits (Table 3). The pattern of appearance of the control and four strains is similar in both barley and chickpea. Control (1) and ΔnifH (5) are close to one another on the far negative quadrant in PC2 (dimension 2), which indicates lack of any influence on the plant traits. *K.o.* M5a1 (4) falls apart from all the vectors on the negative quadrant, however, is still closer to the positive scale, indicating its intermediate effect being higher than control and ΔnifH and lower than other two wild type strains. *K.p.* SSN1 (2) and *K.q.* SGM81 (3) both are positively loaded in PC1 (dimension 1), reflecting maximum positive effect on all the studied parameters. From the spatial distribution of strains obtained here, it is inferred that majority traits are largely affected when plants are treated with *K.p.* SSN1 and *K.q.* SGM81 (Figure 5) and high correlation is established amongst all traits in presence of these strains (Table 3).

## 3. Discussion

We explored the potential of diazotrophic *Klebsiella* strains in plant nutrient (N) uptake and its utilization acting as both biofertilizer and phytostimulator. The results indicate that using *Klebsiella* strains as bioinoculants in barley and chickpea fields improves the plant growth, biochemical traits, and NUE. All the wild type *Klebsiella* strains showed considerable nitrogenase activity (Figure 1). This shows that *Klebsiella* inoculants can be proficiently used as biofertilizer.

*K.p.* SSN1 and *K.q.* SGM81 were found to produce substantial amounts of IAA (57.50 μg mL^−1^ and 85.75 μg mL^−1^ IAA, respectively) using tryptophan as substrate indicating active expression of *ipdC* pathway for IAA production. On the contrary, despite the presence of gene *ipdC*, *K.o.* M5a1 showed no detectable IAA production. The protein sequence of *K.o.* M5a1 *ipdC* gene showed no obvious sequence deviation from *K.q.* SGM81 and *K.p.* SSN1 (including cofactor binding sites, see Supplementary Information S1). The few most common examples of previously reported *Klebsiella* are *Klebsiella* *pneumoniae* producing 22.7 mg L^−1^ of IAA [20] and *Klebsiella* pnb8 producing as high as 869 μg mL^−1^, using plant extract as substrate [21]. IAA is crucial for root development in plants, and IAA-producing microorganisms benefit root architecture in different ways. At phenotypic level, IAA is involved in increasing root hair, promoting adventitious root initiation, bursting out root hair, etc. [22], while at cellular level, in plant cell division, extension, and differentiation, specially of the vascular system of plants [23,24], and hence, enhanced nutrient acquisition [25]. In the present experiments, root phenotype of barley and chickpea was positively affected upon the inoculation of *K.q.* SGM81 and *K.p.* SSN1 above *K.o.* M5a1, favoring over all plant growth and development. This reinforces the potential involvement of IAA in promoting structural morphological changes in the root architecture of *K.q.* SGM81- and *K.p.* SSN1-inoculated plants, and that *K.o.* M5a1 does not contribute to this mechanism in a way that would otherwise be observed due to plant hormone auxin. We were able to relate our findings with some other studies conducted in different crops. Dhungana and Itoh [26] reported *Klebsiella* sp. Sal 1 for increasing fresh root weight in tomato and radish, *K. pneumoniae* demonstrated increase in root length of inoculated moth beans and wheat [20], and *K. variicola* AY-13 for inducing adventitious root initiation in soy bean seedling [27]. These results, along with our observations in barley and chickpea, show the profound role of *Klebsiella* in root development.

PGPB can modify the physiology and functioning of plant parts other than root [22]. Two mechanisms are thought to be accountable for this; first is well documented where plant roots under the influence of PGPB result in enhanced nutrient availability [22]. This will be taken up by aerial parts of the plant leading to modification in the primary metabolism which subsequently results in growth. Second, PGPB by some unknown signal triggers the systemic responses which might lead to plant growth [22]. Due to the former, general phenomenon of nutrient acquisition takes place including nitrogen, phosphorus, and other macro- and micronutrients. Nitrogen availability regulates the C and N in the plant due to the allocation of resources between roots and shoots [28]. In the present study, all the wild type strains improved the overall shoot development compared to control, especially *K.p.* SSN1 (Figure 2). These results are in agreement with Zhu et al. [29], who reported enhanced shoot length and biomass in wheat upon inoculation with PGPR. Experiments conducted by [30] on chickpea in sandy soil resulted into enhanced shoot fresh weight and dry weight in treated plants.

The biochemical parameters (Cl, PR, and NT) that contribute to plant nitrogen were enhanced. The abundance of these biochemical traits in *K.p.* SSN1 and *K.q.* SGM81 above *K.o.* M5a1 clearly demonstrates that coupled effect of root growth-promoting traits (auxin production) and nitrogen-fixing ability of *K.p.* SSN1 and *K.q.* SGM81 on total plant nitrogen is more vital than nitrogen-fixing ability alone of *K.o.* M5a1. This is justifiable on the basis of “multiple mechanism theory” given by Bashan and Levanony [31], which assumes that more than one factor may be responsible for plant growth. Few studies conducted recently emphasize mechanisms other than BNF for improving overall plant nitrogen. For example, Calvo et al. [32] studied different non-nitrogen-fixing *Bacillus* strains which were able to upregulate nitrate and ammonium uptake genes in *Arabidopsis.* Nitrate was being absorbed by the plasma membrane of the cortical and epidermal cells of the roots, thus bringing root structural elements to the frontline [33]. After absorbing and assimilating NO^−^_3_ in the root in some proportion, the remainder was being reduced to ammonium (NH^+^_4_) by nitrate reductase (NR) and transported upwards through the xylem for assimilation in the shoot [34]. Thus, the nitrate reductase present in the plant is proportional to the nitrate and evidence of the high or low plant nitrogen content. As shown in Table 2**,** the result of nitrate reductase assay in barley experiments synchronize with the aforementioned relationship increasing proportionately with increase in plant nitrate.

Improvement in NUE of a plant results in increase of growth yield and decrease in the environmental nitrogen pollution, as suggested by Perchlik and Tegeder [35]. In our study, NUE and its components were increased in both barley and chickpea (Figure 3) upon treatment with wild type strains of *Klebsiella*. We attribute the increase in NUpE to the access of soil nitrogen and hence to the well-developed root system, as hypothesized by Win et al. [36]. This is largely supported by our results of root development in *K.p.* SSN1 and *K.q.* SGM81 plants. Plant growth is associated with nutrient uptake and its proper distribution in the aerial part. Ideally, a plant is considered healthy when, post uptake, it uses the acquired nitrogen dexterously. This depends on genetic construct of a plant and its internal N requirement for metabolism [37]. The NUtE of the treated plants increase compared to control does not show any close correlation with NUE and NUpE (Figure 5). Nitrogen use efficiency not only depends on an efficient N uptake from the soil, but also on the internal transport, storage, recycling, remobilization, and growth stage of the plants [38,39]. Hence, both NUpE and NUtE play important roles in regulating the overall NUE of a plant. Worku et al. [40] showed that in tropical maize hybrids, NUE was influenced by both NUpE and NUtE. However, Rotundo et al. [41], working on soybean, reported that NUpE is more critical than NUtE in determining the increase in NUE. Our results from PCA biplot (Figure 5A,B) clearly indicate that NUpE is closely associated with NUE, and thus is more influential than NUtE in NUE management of both the model plants when treated with diazotroph *Klebsiella*.

## 4. Materials and Methods

### 4.1. Bacteria and Culture Conditions

Three wildtype diazotroph *Klebsiella* strains (*K.p.* SSN1, *K.q.* SGM81, and *K.o.* M5a1) isolated from different rhizosphere soil samples were used as test strains. *K.q.* SGM81 and *K.p.* SSN1 were isolated from the rhizosphere soil of *Dianthus caryophyllus* (carnation) and barley, respectively, from Gujarat, India. The soil source of isolation of *K.o.* M5a1 strain is unknown, however, for current study, it was obtained from Buck Lab at the Department of Life Sciences, Imperial College, London. The whole genome sequence of *K.o.* M5a1 and *K.p.* SSN1 strains were determined, and the bio project was deposited at DDBJ/ENA/GenBank under PRJNA649683 and PRJNA643013, respectively. The details of *K.q.* SGM81 sequence (PRJEB21197) are mentioned in our previous manuscript [9]. *K.q.* SGM81, *K.p.* SSN1, and *K.o.* M5a1 were identified as *Klebsiella quasipneumoniae*, *Klebsiella pneuomoniae,* and *Klebsiella oxytoca,* respectively, based on the average nucleotide identity (ANI)-based taxonomy. A *nifH* mutant of *K.o.* M5a1, a nitrogenase minus variant, was used as a negative control. All the strains were grown in nutrient broth (HiMedia, Ahmedabad, India), unless specifically stated. *K.p.* SSN1 and *K.q.* SGM81 strains were used in the study for their ability to produce plant hormone auxin (IAA) and nitrogenase production. *K.o.* M5a1 was taken as a positive control solely for nitrogenase production, and negative for all other PGPR attributes including IAA. Δ*nifH* was nitrogenase minus strain, and hence a negative control for both IAA and nitrogenase production under nitrogen fixing conditions where it does not grow. These strains were maintained at 4 °C as agar slants until experimental use.

### 4.2. Recombineering M5a1ΔnifH

The Δ*nifH* knockout mutant was derived by Lambda Red recombineering as described by Datsenko and Wanner [42]. Oligonucleotides were designed in order to amplify a curable kanamycin resistance cassette, flanked by Flippase Recognition Targets, (FRT-*nptII*-FRT) from the pGEM-T-KanFRT plasmid [43] with 60 nt overhangs homologous to the flanking regions of the *Ko* M5a1 *nifH* region (pGEM-T-KanFRT binding region in upper case): nifH_mutF, tctgctggcaaacactcaacaacaggagaagtcaccatgaccatgcgtcaatgcgctattGTGTAGGCTGGAGCTGCTTC; nifH_mutR, tggatcagcgccagattacgttcgcccgttgcgttggtcatcataattgtcctgtgctcatccTCCTTAGTTCCTATTCCG. PCR was performed using Phusion polymerase (Thermo Scientific) with 25 ng template plasmid and an annealing temperature of 55 °C. Template DNA was removed by DpnI digestion prior to gel extraction of products. M5a1 was transformed with the pKD46 plasmid, expressing the Lambda Red genes required for homologous recombination under the control of an arabinose-inducible promoter. Competent Red-recombinase expressing cells were prepared in super optimal broth (SOB) (0.5% (*w*/*v*) yeast extract, 2% (*w*/*v*) tryptone, 10 mM NaCl, 2.5 mM KCl, 20 mM MgSO_4_), to which L-arabinose was added to a final concentration of 10 mM once OD_600_ reached 0.1. Cells were grown to an OD_600_ of 0.4–0.6 before being washed three times in sterile cold 10 % glycerol solution. Approximately 1 μg of purified PCR product was incubated with 100 μL of competent cells on ice for 30 min prior to electroporation. Transformed cells were recovered at 30 °C for 3 h before being plated out on LB agar plates supplemented with kanamycin. Kanamycin-resistant colonies were used to generate seed cultures from which genomic DNA was extracted. Locus-specific homologous recombination was confirmed by diagnostic PCR, combining primers specific to regions flanking the knockout locus with those specific to the *nptII* cassette, and Sanger sequencing.

### 4.3. Acetylene Reduction Assay

ARA was carried out to determine the BNF of the isolates following the method described by Kaushal and Kaushal [44]. Briefly, a nitrogen-free Jensen liquid medium was used to inoculate the cultures and was allowed to grow till mid exponential phase at 30 °C for 48 h at 100 rpm. Aliquots with optical density of 0.1 at 600 nm were prepared and used as inoculum for nitrogen-free medium in the air-free assay vials and again incubated till exponential phase was reached. The headspace of the vial was replaced with acetylene (10% *v*/*v*) and incubated for 18 h. After incubation, the gas sample was injected in a Brucker 450 gas chromatograph with flame ionization detector. Calibration was done using standard ethylene gas. Nitrogenase activity was determined in terms of ethylene produced. After ARA, cells were pre-digested, and protein concentration was calculated using Bradford reagent [45]. The nitrogenase activity was calculated using the formula:

Nitrogenase activity (nmole C_2_H_4_ h^−1^ mg protein^−1^) = C × P_s_ × V/P_std_ × T × P (1)

where:

C = concentration of ethylene in ηmoles.
P = protein concentration of bacterial cell in mg.
P_S_ = peak height of sample.
V = volume of air space in the assay vial.
P_Std_ = peak height of standard.
T = time of incubation in h.

### 4.4. IAA Extraction and Determination by Salkowski Reagent Method

IAA production and estimation was carried out following the method given by Gang et al. [46]. IAA biosynthesis in the cultures was induced by supplementing nutrient broth with 0.1% (*w*/*v*) L-tryptophan, and cultures were incubated in the dark at 30 °C on an orbital shaker at 200 rpm. IAA production and secretion were measured in culture supernatants after 24 h using Salkowski reagent. Briefly, 1 mL of culture supernatant was mixed with 1 mL of Salkowski reagent and incubated in the dark for 30 min. Development of pink color was spectrophotometrically measured at 536 nm and IAA quantified using an IAA standard.

### 4.5. Field Experimental Design

#### 4.5.1. Site Description

To understand and evaluate the effect of three different *Klebsiella* strains on the model crop plants barley and chickpea, a full-length seasonal study was conducted in field for respective crops. A site (23.0658 °N, 72.5138 °E) located at Sola Road, Ahmedabad, India, was selected for the field experiments. Both barley and chickpea were sown in the month of November 2019 and harvested in February 2020. The physical characteristics of the soil were recorded as: pH 6.8, organic matter 0.97%, sand 32.2%, silt 35.3%, and clay 29.5%. The nitrogen content of the site before our plantation was 0.83 mg N g^−1^ soil as estimated at Indian Farmers Fertilizer Cooperative Limited (IFFCO)**.** The classification of this soil type is aridisols, according to United States Department of Agriculture (USDA) soil taxonomic system [47].

#### 4.5.2. Plant Treatment and Growth

Certified barley seeds (Karan 201, from Bashino agro India pvt Ltd.), and chickpea seeds (black chickpea, from Gujarat Junagadh gram 3) were purchased. A total of 10 g of seeds (barley and chickpea) for every strain and a control were weighed and sterilized. For sterilizing, the seeds were washed with 70% ethanol for 1 min and subsequently rinsed with 20% sodium hypochlorite thrice, followed by a final wash with sterile distilled water [48]. The sterile seeds were then immersed for 15 min in 0.5% (*w*/*v*) carboxymethyl cellulose (CMC) solution prepared in sterile distilled water to increase the adhesion of bacterial cells on seed surface. The coated seeds were subjected to the bacterization. To prepare the culture for seed bacterization, all strains were inoculated in the sterile nitrogen-free liquid Jensen medium and allowed to grow overnight. After reaching the desired growth at OD_600_ 0.5, cells were pelleted out, washed, and resuspended in 100 mL of 0.6% (*w*/*v*) NaCl so as to remove the traces of nutrient medium. Seeds were soaked in the final prepared culture suspension for 30 min, allowing proper adhesion of the bacterial cells all over the seed surface. For control, seeds were soaked in sterile 0.6% NaCl. Randomized complete block design was used in the field with a split plot arrangement for different treatments. Each sub-plot consisted of prepared seeds for respective treatment. Single seeds were sown at the depth of approximately 1 cm, maintaining the distance of around 15 cm between two neighboring seeds and 25 cm row-to-row spacing. After 15 days of sowing, a booster dose was given to each row. All strains were grown in sterile 1 L nitrogen-free Jensen liquid medium to achieve OD_600_ of 0.5. For booster dose, cells were pelleted to discard any nutrient medium followed by washing and resuspension of the cell pellet in 0.6% (*w*/*v*) NaCl of final volume 1 L. The control set was just supplemented with the same volume of 0.6% NaCl. A complete stepwise description of the process is shown as image schema (Figure S4).

### 4.6. Plant Growth Parameters

Plant traits were studied, with six replicates randomly chosen. The traits were categorized into three sections:

1. Root morphology traits comprising RL, RW, RDW, and NSR. These root traits were measured after uprooting the treated and control plants at the harvest stage after 90 days. Pictures of the roots were taken and processed in Image J for estimating the root diameter. In addition, RSA was calculated using the formula RSA = root length × root diameter × Π.
2. Shoot morphology traits comprising of SL, SW, SDW, and GW per plant.
3. Biochemical traits:

#### 4.6.1. Chlorophyll Content

Cl content was determined following the method given by Arnon [49]. Briefly, 0.5 g fresh leaf was homogenized in 5 mL of 80% acetone and centrifuged at 10,000 rpm for 5 min. The collected supernatant was subjected to optical density measurement at 663 and 645 nm wavelength. Total chlorophyll was calculated as:

Cl (mg mL^−1^) = Cl a + Cl b. (2)

where

Cl a (mg mL^−1^) = 12.7 A663–2.69 A645.
Cl b (mg mL^−1^) = 22.9 A645–4.68 A663.
A645 = absorbance at a wavelength of 645 nm.
A663 = absorbance at a wavelength of 663 nm.

#### 4.6.2. Total Protein

Determination of total soluble PR was done according to Bradford method [50], using albumin bovine. About 0.5 g plant shoot sample was crushed and homogenized in 5 mL phosphate buffer. The homogenized mixture was boiled at 100 °C in water bath for 10 min and centrifuged at 3000 rpm for 5 min. The final reaction consisted of 2 mL d H2O, centrifuged extract (20 μL), and Bradford reagent (0.5 mL). Finally, the optical density was recorded at 595 nm wavelength using a spectrophotometer.

#### 4.6.3. Plant Nitrogen Concentration

The total aboveground nitrogen (ABN) at maturity was measured by Kjeldahl technique, as described by AppliChem [51]. The plants after uprooting were washed thoroughly to get rid of any soil particles. Plant samples were then air-dried for seven days to determine dry weight. The perfectly dried and ground plant was placed in a Kjeldahl digestion flask. To the plant sample, 1 g of catalyst and 5 mL of concentrated H_2_SO_4_ were added. The flask was subjected to heating for 2 h for digestion of the sample and cooled at room temperature. A total of 30 mL of distilled water with four drops of phenolphthalein reagent was added to the flask. N was collected via distillation in 4% (*w*/*v*) boric acid solution. A blank was run without any plant tissue sample. Total %N was determined by titrating the obtained solution with 0.1 M HCl, and expressed as mg N g^−1^ of dry matter.

#### 4.6.4. Nitrate and Nitrate Reductase Activity

A detailed technique for plant nitrate estimation, as described by Zhao and Wang [52], was followed using fresh plant samples. Briefly, fresh plant samples were air-dried in the dark, and aqueous solution was prepared using 1 mL deionized water, centrifuged and supernatant collected. To 0.1 mL of the supernatant, 0.4 mL salicylic acid–sulfuric acid mixture was added and allowed to rest for 20 min at room temperature. A total of 9.5mL of 8% NaOH was added to the above mixture and allowed to cool for 30 min. The final mixture was read at OD_410_. NT concentration was calculated by applying the formula C = 140.86 × OD_410_ − 1.1831. Using the value of C, final plant nitrate content was calculated as Y = CV/W, where Y is nitrate content (μg g^−1^), C is nitrate concentration, V is total volume of extracted sample (mL), and W is weight of sample (g).

For NR activity, 200 mg of fresh plant sample was taken in a vial containing chilled 8 mL of 100 mM phosphate buffer, 0.2 mL of 50 mM KNO_3_, and 1% (*v*/*v*) isopropanol as described by Silveira et al. [53]. The vial was subjected to vacuum infiltration and incubated for 30 min at 30 °C. Vials were then placed in a water bath for 5 min to cease the enzymatic activity. The released nitrite was determined by colorimetric reaction with 1:1 ratio of sulfanilamide prepared in 1 mL of 1 M HCl and 0.02% N-napthyl-ethylene diamine. OD was recorded at 540 nm.

### 4.7. Nitrogen Use Efficiency and Its Components

Upon measuring the plant nitrogen, the following indexes were calculated using the formula given by Moll et al. [54]:

NUpE: *ABN/Ns.*
NUtE: *Gw/ABN.*
NUE: *Gw/Ns.*

Where: ABN is total aboveground nitrogen, *Ns* is available soil nitrogen (0.83 mg g^−1^ soil), and *Gw* is grain weight.

### 4.8. Statistical Analysis

A two-way ANOVA and descriptive analysis were performed to analyze the effect of BNF and IAA by strains on nitrogen use efficiency of the barley and chickpea using GraphPad Prism 8.0. Level of significance was studied for mean comparison at 5% significance level. The data is represented as mean ± standard error (SE) of six replicates. PCA was performed in R (3.6) using strains as individuals and traits as variables. The contribution of each measured and calculated trait towards total variability was established by PCA. The correlation of each variable and individual with the principal components was also generated to understand their contribution.

## 5. Conclusions

Being a highly nutritious and staple food of large populations, barley and chickpea are among the world’s most grown crops, and hence the contributors to food nitrogen footprint. The field experiments demonstrate the effectivity of the three tested diazotrophs *klebsiella quasipneumoniae* SGM81, *Klebsiella pneumoniae* SSN1, and *Klebsiella oxytoca* M5a1 in significantly increasing the nitrogen uptake and use efficiencies and overall improvement in morphology and biochemical parameters of both the crops, however still maintaining the order *K.p.* SSN1 ≥ *K.q.* SGM81 > *K.o.* M5a1. This order difference could be attributed to expression of a single plant beneficial trait (BNF) in *K.o.* M5a1, yet more than one (IAA and BNF) in *K.p.* SSN1 and *K.q.* SGM81. The enhanced ability for nitrogen utilization in plants could perhaps be facilitated by using molecular biotechnology tools to improve genes of the plant system. We further conclude that *K.p.* SSN1 is a strong candidate for bioinoculation for barley and chickpea in agriculture. For application on a wide variety of crops and vegetables, the effects of other plant growth-promoting attributes of *K.p.* SSN1 and *K.q.* SGM81, such as phosphate solubilization, siderophore production, etc., could be explored and scaled from in vitro to field trials.

## Figures and Tables

**Table 1:** Measurements of root morphology traits in barley and chickpea plants.

Plants	Traits	Treatment				
**Barley**		Control	*K.p.* SSN1	*K.q.* SGM81	*K.o.* M5a1	*Δnif H*
	Root Fresh weight (g)	1.5 ± 0.039 ***	6.58 ± 0.0084 ***	5.117 ± 0.060 ***	1.84 ± 0.0093 ***	1.205 ± 0.085 ***
	Root dry weight (g)	0.8 ± 0.050 ***	4.217 ± 0.1249 ***	3.4 ± 0.057 **	0.9200 ± 0.01125 ***	0.300 ± 0.0057 ***
	Root length (cm)	8.00 ± 0.392 ***	22.12 ± 0.1195 ***	18.08 ± 0.149 ***	10.00 ± 0.1493 ***	6.00 ± 0.0077 ***
	Root diameter (mm)	9.583 ± 0.1417 *	25.75 ±.6180 ***	18.42 ± 0.3780 ***	15.94 ± 0.5707 ***	7.304 ± 0.3696 *
	Root surface area(cm)	220.7 ± 16.02 **	1796 ± 31.35 ***	1062.2 ± 22.90 ***	494.5 ± 29.47 ***	136.2 ± 88.39 ^ns^
	Number of secondary roots	10.33 ± 0.88 **	18.67 ± 1.202 **	22.00 ± 0.57 **	11.00 ± 0.5774 ^ns^	8.33±0.33 ^ns^
**Chickpea**	Root Fresh weight (g)	1.117 ± 0.060 **	3.283 ± 0.070 ***	2.5 ± 0.03 **	1.735 ± 0.04 *	0.89 ± 0.008 *
	Root dry weight (g)	0.42 ± 0.012 **	1.78 ±0.018 ***	1.43 ± 0.0095 ***	0.52 ± 0.0079 ***	0.266 ± 0.006 ***
	Root length (cm)	7.117 ± 0.060 ***	19.82 ± 0.1302 ***	18.68 ± 0.364 ***	8.017 ± 0.1108 **	7.033 ± 0.066 ^ns^
	Root diameter (mm)	10.26 ± 0.765 *	12.72 ± 0.459 ^ns^	11.18 ± 0.415 ^ns^	13.66 ± 0.6018 **	12.74 ± 0.4287 *
	Root surface area (cm)	226.3 ± 15.41 **	802.7 ± 26.66 ***	676.2 ± 13.93 ***	350.4 ± 16.50 **	276.0 ± 10.55 ^ns^
	Number of secondary roots	3.33 ± 0.88 ^ns^	5.00 ± 0.5 ^ns^	5.33 ± 0.3 ^ns^	3.33 ± 0.3 ^ns^	4.00 ± 0.57 ^ns^

Effect of five treatments on root architecture of barley and chickpea. Statistical analysis was done using GraphPad Prism 5 software calculated at *p* ≤ 0.05. Data represents mean values of six replicates with standard error. Significance of data was analyzed using ANOVA and mentioned through ‘*’. Key: * (*p* ≤ 0.05), ** (*p* ≤ 0.01), *** (*p* ≤ 0.001), ^ns^ non-significant.

**Table 2:** Measurements of biochemical traits in barley and chickpea plants.

Plants	Traits	Treatment				
**Barley**		Control	*K.p.* SSN1	*K.q.* SGM81	*K.o.* M5a1	*Δnif H*
	Chlorophyll (mg mL^−1^)	1.079 ± 0.0547 *	1.530 ± 0.1281 **	1.372 ± 0.110 *	1.362 ± 0.0614 *	1.165 ± 0.04829 *
	Protein (mg g^−1^ FW h^−1^)	1.200 ± 0.0774 *	2.130 ± 0.1134 **	2.042 ± 0.1428 **	1.395 ± 0.1919 **	1.133 ± 0.1436 ^ns^
	Nitrate reductase (µg g^−1^ FW h^−1^)	10.22 ± 0.3156 **	14.20 ± 0.5447 ***	14.60 ± 0.2781 ***	10.93 ± 0.6344 *	10.12 ± 0.4257*
	Nitrate (µg g^−1^)	381.2 ± 450.0**	604.2 ± 37.93***	593.5 ± 33.25***	424.2 ± 12.60^ns^	395.1 ± 22.26 ^ns^
	Total nitrogen (mg g^−1^)	22.16 ± 2.482*	34.81 ± 3.875**	38.16 ± 1.447**	29.66 ± 4.425*	21.35 ± 1.384 ^ns^
**Chickpea**	Chlorophyll (mg mL^−1^)	0.817 ±0.07417 ***	1.685 ± 0045 **	1.450 ± 0.065 **	1.222 ± 0.1756 **	0.8275 ±0.060 *
	Protein (mg g^−1^ FW h^−1^)	1.295 ± 0.1350 *	1.760 ± 0.1395 *	1.967 ± 0.0928 **	1.577 ± 0.0954 ^ns^	1.433 ± 0.0954 ^ns^
	Nitrate reductase (µg g^−1^ FW h^−1^)	10.55 ± 0.4796 **	15.53 ± 1.322 ***	16.57 ± 0.9735 ***	17.35 ± 0.6020 ^ns^	13.67 ± 0.2257 ^ns^
	Nitrate (µg g^−1^)	353.1 ± 17.83 **	533.0 ± 34.62 **	484.2 ± 7.955 **	484.9 ± 17.21 ^ns^	397.3 ± 6.172 ^ns^
	Total nitrogen (mg g^−1^)	13.65 ± 2.724 **	39.00 ± 1.483 ***	33.00 ± 3.183 ***	28.50 ± 1.310 **	11.83 ±5.084 ^ns^

Effect of five treatments on biochemical traits of barley and chickpea. Statistical analysis was done using GraphPad Prism 5 software calculated at *p* ≤ 0.05. Data represents mean values of six replicates with standard error. Significance of data was analyzed using ANOVA and mentioned through ‘*’. Key: * (*p* ≤ 0.05), ** (*p* ≤ 0.01), *** (*p* ≤ 0.001), ^ns^ non-significant.

**Table 3:** Correlation of the plant traits (variables) of barley and chickpea with the principal components dimension 1 and dimension 2.

Trait	Barley		Chickpea	
	Dim.1	Dim.2	Dim.1	Dim.2
Root fresh weight (RFW)	0.9801	−0.1458	0.9817	0.0430
Root length (RL)	0.9897	−0.0874	0.9480	0.3087
Shoot length (SL)	0.9713	0.0506	0.9721	0.1266
Total nitrogen (Nt)	0.9438	0.0923	0.9705	−0.2037
Chlorophyll (Cl)	0.9130	0.2394	0.9893	−0.1326
Nitrate reductase (NR)	0.9650	−0.1917	0.6626	−0.5968
Nitrate (NT)	0.9664	−0.2451	0.9306	−0.3403
Protein (PR)	0.9918	−0.1094	0.8870	0.0474
Number of secondary roots (NSR)	0.9806	−0.1137	0.7895	0.4932
Nitrogen use efficiency (NUE)	0.9776	0.1497	0.9938	0.1065
Nitrogen uptake efficiency (NUpE)	0.9632	0.2575	0.9705	−0.2037
Nitrogen utilization efficiency (NUtE)	−0.2301	−0.9407	−0.2182	0.7675
Grain weight (GW)	0.9774	0.1509	0.9945	0.1042
Root diameter (Rdia)	0.9629	0.1877	0.2290	−0.7412
Root surface area (RSA)	−0.1874	0.9773	0.9815	0.1875
Root dry weight (RDW)	0.9760	−0.1830	0.9616	0.2426
Shoot dry weight (SDW)	0.9821	0.0292	0.9883	0.0800

## Data Availability

The datasets generated and analyzed during the current study are available in the DDBJ/ENA/GenBank under the following accession numbers: *K.o.* M5a1-PRJNA649683 (https://www.ncbi.nlm.nih.gov/bioproject/?term=PRJNA649683, accessed on 1 February 2021) *K.p.* SSN1-PRJNA643013 (https://www.ncbi.nlm.nih.gov/bioproject/?term=PRJNA643013, accessed on 1 February 2021) *K.q.* SGM81-PRJEB21197 (https://www.ncbi.nlm.nih.gov/bioproject/?term=PRJEB21197, accessed on 1 February 2021).

## Supplementary Materials

The following are available online at https://www.mdpi.com/article/10.3390/plants10040780/s1, Figure S1: Multiple sequence alignment, Figure S2: Promoters of *ipdC* in SGM81 and M5a1, Figure S3: Plant morphology of barley and chickpea, Figure S4: Schematic representation of plant experimental set up.
