# Supplementary material for: Bacterial Indole-3-Acetic Acid Influences Soil Nitrogen Acquisition in Barley and Chickpea
Source: Plants (Basel). 2021 Apr 16;10(4):780. doi: 10.3390/plants10040780 (PMC8071533; doi:10.3390/plants10040780)
Supplement: Supplementary file 1 [file plants-10-00780-s001.zip › plants-1113772-SI.pdf]

Supplementary data

```
M5a1        MQPTYTIGDYLLDRLVDCGIDRLFGVPGDYNLQFLDSVIAHRNLGWVGCANELNAAYAAD 60
SGM81       MQPTYTIGDYLLDRLVDCGIDRLFGVPGDYNLQFLDRVIAHNALGWVGCANELNAAYAAD 60
SSN1        MQPTYTIGDYLLDRLVDCGIDRLFGVPGDYNLQFLDRVIAHNALGWVGCANELNAAYAAD 60
            ************************************ ****. *****************

M5a1        GYARIKGAGALLTTYGVGELSALNGIAGSYAEHVPVLHIVGAPCTGAQQRGELLHHTLGD 120
SGM81       GYARIKGAGALLTTYGVGELSALNGVAGSYAEHIPVLHIVGAPSTGAQQRGELLHHTLGD 120
SSN1        GYARIKGAGALLTTYGVGELSALNGVAGSYAEHIPVLHIVGAPSTGAQQRGELLHHTLGD 120
            *************************:********:*********.***************

M5a1        GDFSHFSRMSEQITCSQAVLAAGNACHEIDRVLSEMLTHHRPGYLMLPADVAKAKTTPPA 180
SGM81       GDFRHFARMSEQITCSQALLTAGNACHEIDRVLRDMLTHHRPGYLMLPADVARAAAIAPA 180
SSN1        GDFRHFARMSEQITCSQALLTAGNACHEIDRVLRDMLTHHRPGYLMLPADVARAAAIAPA 180
            *** **:***********:*:************ :*****************:* :  **

M5a1        HRLLIQGLPADENQLAGFREHAGRMLRSSRRVSLLADFLAQRYGLQNALREWVAKVPVAY 240
SGM81       QRLLVEAAPADENQFAGFCEHASRLLRGSRRISLLADFLAQRYGLQNTLREWVAKTPVAH 240
SSN1        QRLLVEAAPADENQLAGFCEHASRLLRGSRRISLLADFLAQRYGLQNTLREWVAKTPVAH 240
            :***::. ******:*** ***.*:**.***:***************:*******.***:

M5a1        ATMLMGKGLFDEQQSGFVGTYSGIASAEETRDAIENADTIICIGTRFTDTITAGFTQHLP 300
SGM81       ATMLMGKGLFDEQQRGFVGTYSGIASAPQTREAIENADTIICIGTRFTDTITAGFTQHLA 300
SSN1        ATMLMGKGLFDEQQRGFVGTYSGIASAPQTREAIENADTIICIGTRFTDTITAGFTQHLA 300
            ************** ************ :**:***************************

M5a1        LEKTIEIQPFAVRVADRWFSRIPMEKALAILIELSASLAAEWVSPNIQAPGVSGAPEGSL 360
SGM81       RDKTIEIQPFAVRVGDHWFSGVPMDQALAALMTLSAPLAAEWAAPQVVAPEVEEGADGEL 360
SSN1        RDKTIEIQPFAVRVGDHWFSGVPMDQALAALMTLSAPLAAEWAAPQVVAPEVEEGADGEL 360
             :************.*:*** :**::*** *: *** *****.:*:: ** *. . :*.*

M5a1        TQKNFWNTVQKQLRPGDIILADQGTAAFGAAALKLPVDATLIVQPLWGSIGFTLPAAYGA 420
SGM81       TQKNFWATVQGALRPGDIILADQGTAAFGIAALKLPSEASLIVQPLWGSIGFTLPAAYGA 420
SSN1        TQKNFWATVQGALRPGDIILADQGTAAFGIAALKLPSEASLIVQPLWGSIGFTLPAAYGA 420
            ****** ***  ***************** ****** :*:********************

M5a1        QIAAAERRVVLIVGDGAAQLTIQELGSMLRDKQRPLILLLNNEGYTVERAIHGPEQRYND 480
SGM81       QTAAAERRVVLIVGDGAAQLTIQEMGSMLRDKQKPLILLLNNEGYTVERAIHGPEQRYND 480
SSN1        QTAAAERRVVLIVGDGAAQLTIQEMGSMLRDKQKPLILLLNNEGYTVERAIHGPEQRYND 480
            * **********************:********:**************************

M5a1        IALWDWNRLPDAFAPDVPSRCWRVTRTQELQEAMNSSVASDRLTLVEVMLPKMDIPDFLR 540
SGM81       IALWDWRRLPEAFAPDVASRCWRVTHTDELREAMAESITSDMLTLVEVMLPKMDIPDFLR 540
SSN1        IALWDWRRLPEAFAPDVASRCWRVTHTDELREAMAESITSDTLTLVEVMLPKMDIPDFLR 540
            ******.***:****** *******:*:**:*** .*::** ******************

M5a1        TVTQALEERNSRV        553
SGM81       AVTQALEERNSRV        553
SSN1        AVTQALEERNSRV        553
            .************
```

Figure S1. Multiple Sequence alignment of ipdC derived amino acid sequence of indole pyruvate decarboxylase of *Klebsiella* SGM 81, *Klebsiella* SSN1 and *Klebsiella* M5a1, using Clustal O. Highlighted motifs (yellow) and residues involved in Mg^{2+} binding (red), thiamine pyrophosphate binding (blue highlighted) are identical to those of the functional IpdC of *Enterobacter cloacae*, for which the structure and function has been detailed [1][3]

>SGM81 ipdC promoter seq (400nt before the translational start codon ATG)
CCGCCGAGCCCGGCGGCGGCCCATAGTTGTTGGCCAGGTCGAAATGGGTAATGCCGAGGTCA
AACGCGTGCTGGAGCATCTGCCGGCTGGTTTCCACCCGCGTTTCATCGCCAAAGTTATGCCAC
AGACCGAGAGAAATTGCCGGCAGCTGAAGGCCGCTGCGGCCGCAGCGACGATAGGCCATCG
TTTGATATCGCGTGGTACTTGCCTGGTAAACATTCTTCCTCCGCAGATAAAACAGGGTGTGTA
TACGTTTACACTAATCAATGTTCTGCGTGGAGTACATCGCTTTTTCGGCGTAATTATTGCTTTA
CACCGCCTTTATCACCCCCTTTCTGGACAGGCGGATCGCTTATTCAATACTCAAATTACACCT
GCGCCAAAATGAGGAAAATGGAT

>M5a1 ipdC promoter seq (400nt before the translational start codon ATG)
CTCTCCGCCGATCCCGGCGGTGGCCCGTAGTTATTGGCTAAATCGAAATGGGTTATGCCGAGA
TCGAATGCGTGACGCAGAAGCTGACGGCTGGTTTCCACCCGCGTTTCATCGCCAAAATTATGC
CAAAGCCCCAGAGAAACTGCCGGTAGCTTTAGTCCGCTATGACCGCAGCGGCGGTATTCCAT
CGTTTGATATCGCGTGGCGTTTGCCTGGTAAACCATACTTCCTCCGCAGATAAAACAGGGTTG
TGTATACGTTTACACTAAAGAATCTTTGGCGCCGAGTATACTCCCTGAACGCGTAAATTTTAC
TTGCCACCGCATTTGTTGGGTGCAATGTGGACAGCGCAACTGCTTATTCAATACTCAACTTAT
CTGCATGAGAGGAGAGTGGGGAT

Figure S2. Inspection of potential s^{54} promoters of *ipdC* in SGM81 and M5a1, 400 nucleotides before the translational start codon ATG. SGM81 has two near perfect s^{54} promoters with regards to the near invariable putative -24 GG and the -12 GC motifs (red) and their conserved next neighbour nucleotides according to Barrios et al. [2](T at – 25, CA at -22-21, TT at -14-13). Importantly the exact spacing of 10 nucleotides between the -24 GG and -12 CC is stringent for s^{54} recognition and has been validated experimentally to be required for various s^{54} dependent promoters [3]. The spacing between the two putative GG – GC motifs of M5a1 (red) are 9 and 11 nucleotides, suggesting these are pseudo s^{54} binding motifs.

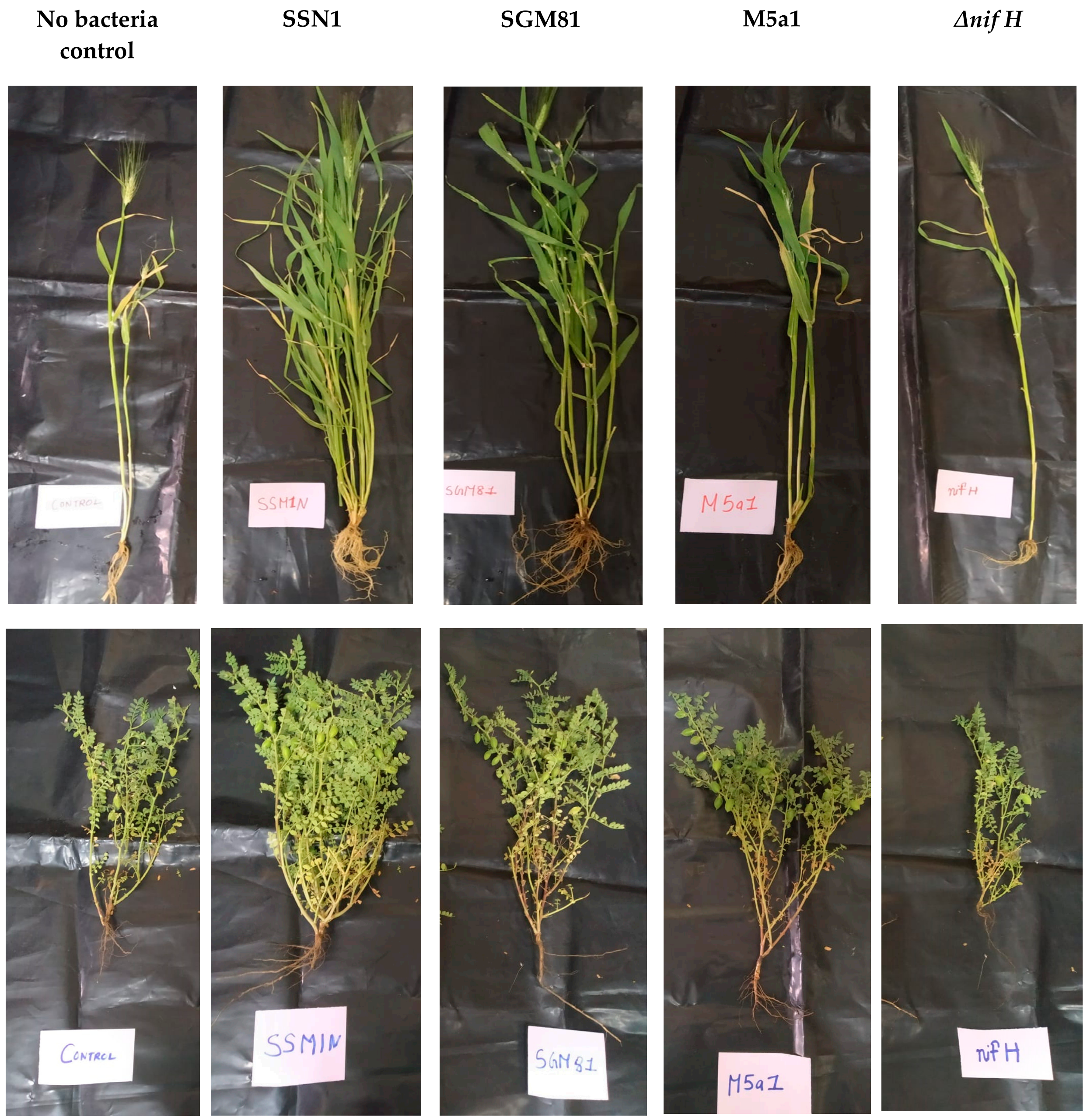

Figure S3. Plant morphology of barley **(A)** and chickpea **(B)** From left to right, no bacteria control, SSN1, SGM81, M5a1, *Δnif H* treatments

Supplementary Fig S4. Image Schema

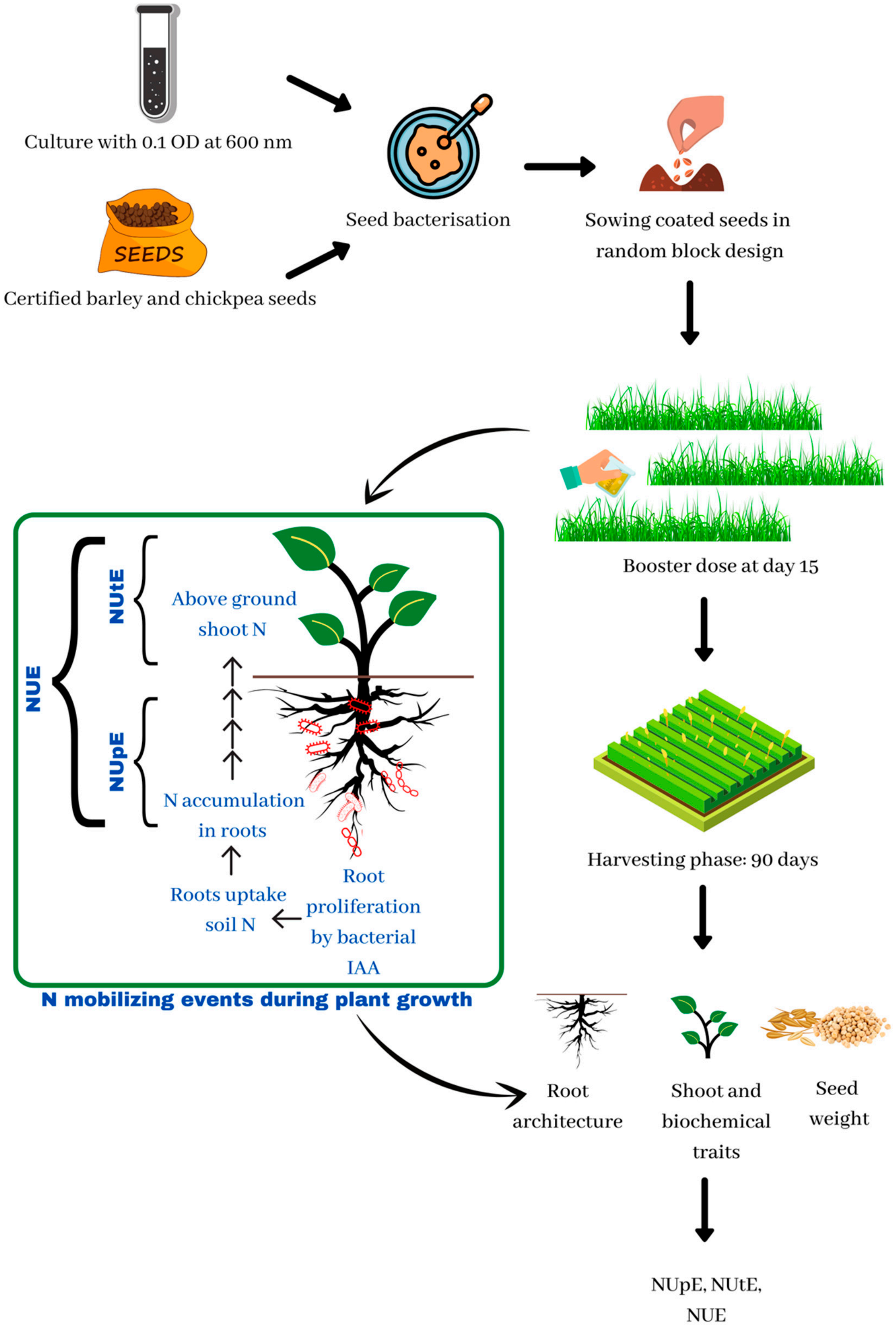

Supplementary Fig S4. Schematic representation of plant experimental set up.

Referen

Flow chart shows the steps conducted for plant study

1. Schütz, A.; Sandalova, T.; Ricagno, S.; Hübner, G.; König, S.; Schneider, G. Crystal Structure of Thiamindiphosphate-dependent Indolepyruvate Decarboxylase from Enterobacter Cloacae, an Enzyme Involved in the Biosynthesis of the Plant Hormone Indole-3-acetic Acid. *Eur. J. Biochem.* **2003**, *270*, 2312–2321.
2. Barrios, H.; Valderrama, B.; Morett, E. Compilation and Analysis of Σ54-Dependent Promoter Sequences. *Nucleic Acids Res.* **1999**, *27*, 4305–4313.
3. Buck, M. Deletion Analysis of the Klebsiella Pneumoniae Nitrogenase Promoter: Importance of Spacing between Conserved Sequences around Positions-12 and-24 for Activation by the NifA and NtrC (GlnG) Products. *J. Bacteriol.* **1986**, *166*, 545–551.
